# Universal administration of nirsevimab in infants: an analysis of hospitalisations and paediatric intensive care unit admissions for RSV-associated lower respiratory tract infections

**DOI:** 10.1007/s00431-025-06125-5

**Published:** 2025-05-16

**Authors:** Lorena Bermúdez-Barrezueta, Vanesa Matías del Pozo, José Manuel Marugán-Miguelsanz, Elena Infante López, Pilar Uribe-Reina, Yara Romero del Hombrebueno, Antonio Jesús Morales-Moreno, Silvia Rojo-Rello, José María Eiros, María Asunción Pino-Vázquez

**Affiliations:** 1 Department of Paediatrics, Faculty of Medicine, Valladolid University, Valladolid, Spain; 2 División of Paediatric and Neonatal Intensive Care, Department of Paediatrics, Hospital Clínico Universitario de Valladolid, Valladolid, Spain; 3 Division of Neonatology, Department of Paediatrics, Hospital Clínico Universitario de Valladolid, Valladolid, Spain; 4 Division of Gastroenterology and Paediatric Nutrition, Head of Department of Paediatrics, Hospital Clínico Universitario de Valladolid, Valladolid, Spain; 5 Department of Paediatrics, Hospital Clínico Universitario de Valladolid, Valladolid, Spain; 6 Microbiology and Immunology Department, Hospital Clínico Universitario de Valladolid, Valladolid, Spain; 7 Head of the Microbiology Department, Professor of Microbiology, Hospital Universitario Rio Hortega, University of Valladolid, Valladolid, Spain

**Keywords:** Lower respiratory tract infection, Nirsevimab, Paediatric intensive care unit, Respiratory syncytial virus

## Abstract

The aim of this study was to assess the impact of universal nirsevimab administration on hospitalisations and paediatric intensive care unit (PICU) admissions due to lower respiratory tract infection associated with respiratory syncytial virus (RSV-LRTI). An observational study was conducted at a tertiary hospital in Spain to compare the frequency and characteristics of children under five years of age hospitalised for RSV-LRTI between October 2023 and March 2024 (nirsevimab period), with the data from two prepandemic COVID- 19 seasons (2018–2019 and 2019–2020) and one postpandemic season (2022–2023). A total of 311 patients were included in the study. During the nirsevimab period, a decrease in the number of children hospitalised for RSV-LRTI was observed, particularly for children under six months of age. Compared with the prepandemic period, there was an 83.3% decrease in hospitalisations and a 73.3% reduction in PICU admissions in this age group. Similarly, compared with the postpandemic period, there was a 90.8% reduction in hospitalisations and an 87.9% reduction in PICU admissions. Furthermore, the median age was greater (15.6 months; IQR 11.1–27.3) than it was in the prepandemic period (4 months; IQR 1.6–8.9) and postpandemic period (3.4 months; IQR 1.5–10.6) (*p* < 0.001). Moreover, the length of hospital stay during the nirsevimab period (4 days; IQR 3–6) was shorter than that observed during the prepandemic period (6 days; IQR 4–9) and the postpandemic period (5 days; IQR 3–8) (*p* = 0.003).

*Conclusions*: Following the introduction of universal immunoprophylaxis with nirsevimab, notable reductions in hospitalisations and PICU admissions due to RSV-LRTI were observed among young infants. This resulted in a shift in the age profile and a shorter length of hospital stay.

**Table:** 

**What Is Known** • Nirsevimab is a novel humanised IgG1 monoclonal antibody with a prolonged half-life that has been demonstrated to reduce RSV-associated hospitalisations in controlled clinical trials; however, real-world data are still limited.	
**What Is New:** • The findings of the present study corroborate the effectiveness of nirsevimab. Following the implementation of universal immunoprophylaxis with nirsevimab, a notable reduction in hospitalisations and admissions to the paediatric intensive care unit for RSV-associated lower respiratory tract infections was observed, particularly among infants younger than 6 months, who have been the main target of this passive immunisation strategy. In addition, the patients admitted were older and the length of hospital stay was shorter.

**What Is Known**

• Nirsevimab is a novel humanised IgG1 monoclonal antibody with a prolonged half-life that has been demonstrated to reduce RSV-associated hospitalisations in controlled clinical trials; however, real-world data are still limited.

**What Is New:**

• The findings of the present study corroborate the effectiveness of nirsevimab. Following the implementation of universal immunoprophylaxis with nirsevimab, a notable reduction in hospitalisations and admissions to the paediatric intensive care unit for RSV-associated lower respiratory tract infections was observed, particularly among infants younger than 6 months, who have been the main target of this passive immunisation strategy. In addition, the patients admitted were older and the length of hospital stay was shorter.

## Introduction

Lower respiratory tract infections (LRTIs) are common in infancy and can sometimes be severe. Respiratory syncytial virus (RSV) is the primary aetiological agent and the main cause of hospitalisation in infants, often leading to acute respiratory failure that requires care in a paediatric intensive care unit (PICU) [1, 2]. It is estimated that each year, RSV causes 33 million LRTIs worldwide in children under five years of age, resulting in 3.6 million hospitalisations and 101,000 deaths [3].

There is no specific drug for the treatment of RSV infections, and immunoprophylaxis with palivizumab has been the only strategy used for many years during peak RSV seasons in certain high-risk groups [4, 5]. Nirsevimab is a new humanised IgG1 monoclonal antibody designed to target the pre-F protein of RSV. This long-acting monoclonal antibody blocks viral entry and has an extended half-life, providing at least 150 days of protection [6]. The European Medicines Agency (EMA) in October 2022 and the United States Food and Drug Administration (FDA) in July 2023 approved its use in newborns and healthy infants to prevent severe RSV infection in the first season [7, 8]. In Spain, universal immunoprophylaxis with nirsevimab was promoted during the 2023–2024 epidemic season [9, 10]. Specifically, in the Castilla y León region, passive immunisation with this monoclonal antibody was introduced among all newborns from 1 October 2023. Furthermore, its administration is indicated for infants under six months of age facing their first RSV season, in addition to its use in vulnerable children under two years of age [11]. The aim of this study was to assess the impact of universal nirsevimab administration on the incidence of hospitalisations and PICU admissions due to RSV-associated lower respiratory tract infection (RSV-LRTI) in a health area in Spain.

## Methods

An observational study was conducted at the Hospital Clínico Universitario de Valladolid, a tertiary care hospital of the public health system located in the Castilla y León region of north-central Spain. The hospital provides health care to the Valladolid-East health area, and its PICU admits patients transferred from other second-level hospitals in the locality and from other provinces, including Segovia and Palencia. The PICU provides health care coverage to approximately 95,000 children under 14 years of age, 27,000 of whom are under five years of age [12–14]. The birth rate has remained stable over the last 6 years, with approximately 1,000–1,100 births per year [15].

From 1 October 2023 to 31 March 2024, nirsevimab immunoprophylaxis was administered to all newborns with a gestational age of 35 weeks or above at 48 h of age prior to maternal discharge, following parental consent. Furthermore, infants under six months of age at the start of the epidemic season (born between 1 April and 30 September 2023) and high-risk children under two years of age received a dose at primary care centres within the hospital's health care area. The paediatric population at high risk of severe RSV disease can be divided into the following groups [10]:

1. Preterm infants with a gestational age of less than 35 weeks during their first year of life.
2. Patients with congenital heart disease with significant haemodynamic impairment.
3. Bronchopulmonary dysplasia.
4. Other underlying conditions, including severe immunodeficiency, inborn errors of metabolism, cystic fibrosis, neuromuscular disease, Down syndrome, and other genetic syndromes with relevant respiratory problems.

During the nirsevimab immunisation campaign, a total of 1,075 infants received nirsevimab in the hospital's health area, of whom 450 received it at maternity discharge and 625 at primary care centres. Local data indicate that nirsevimab coverage in the designated health area was 94.7% for infants born during the epidemic season and 92.9% for those born from 1 April to 30 September 2023 [16].

### Study population

The study population included children under five years of age who were hospitalised due to RSV-LRTI. Patients with positive RSV detection but without symptoms of LRTI were excluded from the study. LRTIs are defined as infections that affect the airways below the larynx, including the trachea, bronchi, bronchioles, and alveoli. These infections include conditions such as pneumonia, bronchitis, and bronchiolitis, which are characterised by symptoms such as tachypnoea, dyspnoea and acute respiratory failure with low oxygen saturation (SpO2) or hypercapnia in severe cases, with or without fever [1].

Patients hospitalised between October 2023 and March 2024 who met the inclusion criteria were prospectively enrolled in the study (i.e., the nirsevimab period). We compared the frequency of admissions and the characteristics of patients in the nirsevimab period with the same data from the two prepandemic epidemic seasons (2018–2019 and 2019–2020) and the postpandemic season (2022–2023). Each epidemic season spanned from October to March of the following year. Given the observed variation in RSV epidemiology during the ongoing pandemic [17, 18], patients admitted during the 2020–2021 and 2021–2022 seasons were excluded from the present analysis.

During the study period, the aetiology was investigated in nasopharyngeal swabs or tracheal aspirate samples via molecular diagnostic tests (Luminex NxTAG Respiratory Pathogen Panel or FilmArray® Respiratory Panel). The following agents were identified: RSV, adenovirus, coronaviruses (229E, HKU1, OC43, NL63 and MERS), human metapneumovirus, enterovirus/rhinovirus, and influenza A (A/H1 - 2009, A/H3), influenza B, parainfluenza (1, 2, 3, 4), *Chlamydia pneumoniae*, *Mycoplasma pneumoniae*, *Bordetella pertussis, Bordetella parapertussis* and *Legionella pneumophila*. During the study periods, the molecular tests used did not change. Additionally, since the COVID- 19 pandemic, RT‒PCR has been used for the detection of SARS-CoV- 2.

### Data Collection

The dataset included a range of data, including demographic, clinical, and laboratory information, as well as data on viral coinfections, treatments, respiratory support, the necessity for PICU admission, the length of hospitalisation, and the length of stay in the PICU.

Clinical data concerning patients in the pre- and postpandemic periods were collected retrospectively via JIMENA 4.0 software, which is the medical records management tool of the SACYL (Castilla y León Health Service). An exhaustive search for diagnoses, including acute respiratory failure, acute lower respiratory tract infections, bronchiolitis, bronchitis, bronchospasm and pneumonia, was performed.

### Ethical considerations

The study was approved by the Research Ethics Committee of the Valladolid Health Department (internal code 23–3377) in accordance with the regulations of the 1964 Declaration of Helsinki and its later amendments. During the prospective phase, patients were enrolled after providing informed consent from their parents or legal guardians.

### Statistical analyses

Statistical analysis was performed with IBM SPSS Statistics 29.0.1.0 (IBM Corp., Armonk, New York, NY, USA). Categorical variables are expressed as absolute values and percentages with 95% confidence intervals (95% CIs), whereas quantitative variables are expressed as medians and interquartile ranges (IQRs). Continuous variables were analysed via the Kruskal‒Wallis test, whereas categorical variables were analysed using either Fisher's exact test or Pearson's chi‒square test. A p value of less than 0.05 was considered statistically significant.

## Results

**Fig. 1:**
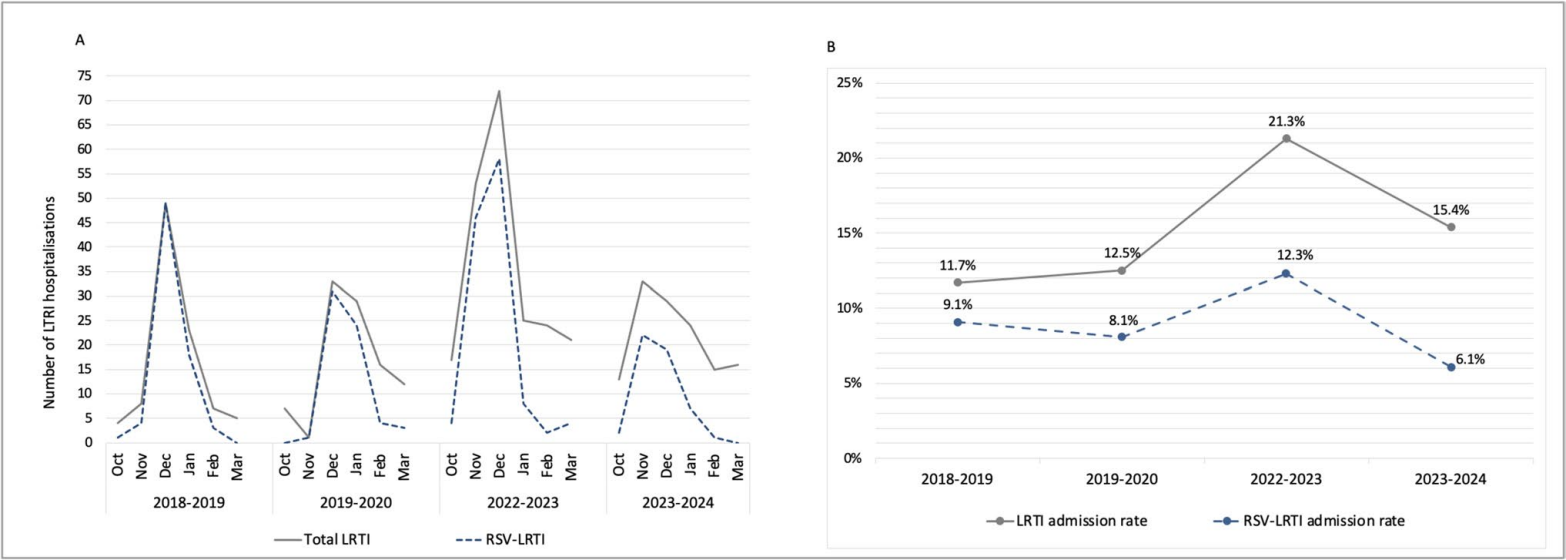
**A.** Monthly distribution of total hospitalisations for LRTI and RSV-associated LRTI in children under 5 years of age by epidemic season. **B.** Rates of LRTI and RSV-LRTI hospitalisations by epidemic season

A total of 536 children under the age of five were hospitalised for LRTI over the course of the three study periods, with 58% of these cases being associated with RSV. During the prepandemic period, RSV was responsible for 71.1% (95% CI 64.5–77.8) of hospitalisations for LRTI, a figure that decreased to 57.5% (95% CI 50.7–64) in the postpandemic period and further decreased to 39.2% (95% CI 30.5–48) during the nirsevimab period (*p* < 0.001). The RSV-LRTI admission rate decreased significantly during the nirsevimab period relative to the prepandemic period (8.6% vs. 6.1%; *p* = 0.025) and the postpandemic period (12.3% vs. 6.1%; *p* < 0.001). This rate was calculated by dividing the number of RSV-LRTI hospitalisations by the total number of admissions to the paediatric department during each epidemic season. The monthly distributions of LRTI and RSV-LRTI hospitalisations and incidence rates during each epidemic season are shown in Fig. 1.

The present study analysed 311 cases of RSV-LRTI. The median age was 4.9 months (IQR 1.7–13.7), and the median weight was 7 kg (IQR 4.8–10). The study population was 57.5% male and 53.7% less than six months of age. The primary diagnoses were bronchiolitis (63.9%), bronchospasm or bronchitis (28.1%), and pneumonia (7.7%). Table 1 presents the characteristics of the study population.

**Table 1:** Demographic and clinical characteristics of children hospitalised due to RSV-associated LRTI

	*n *= 311 (%)
Age (months); *median [IQR]*	4.9 [1.7–13.7]
Weight (kg); *median [IQR]*	6.9 [4.7–9.9]
Males; *n* (%)	179 (57.6)
Gestational age (weeks)	39 [38–40]
Prematurity; n (%)	41 (13.2)
Patients with at least 1 comorbidity; n (%)	26 (8.4)
^a^Underlying disease; n (%) Cardiopathy Bronchopulmonary dysplasia Cystic fibrosis Neuromuscular disease Congenital malformation syndromes Down's syndrome Other	9 (2.9) 4 (1.3) 1 (0.3) 9 (2.9) 6 (1.9) 1 (0.3) 4 (1.3)
Diagnosis; n (%) Bronchiolitis Bronchospasm Pneumonia Whooping cough	199 (63.9) 87 (27.9) 24 (7.7) 1 (0.3)
Apnea	18 (5.8)
C-reactive protein mg/L (*n* = 263); *median [IQR]*	16.2 [3.8–50]
*Bacterial infection	27 (8.9)
Antibiotic therapy	109 (35)
Viral coinfection	180 (57.9)
Viral Coinfections RSV-Rhino/enterovirus RSV-Coronavirus (229E, HKU1, OC43, NL63) RSV-Bocavirus RSV-Parainfluenza 1- 4 RSV-Adenovirus RSV-Influenza (A, A/H1, A/H1 - 2009, A/H3, B) RSV-Metapneumovirus RSV-SARS-CoV- 2	108 (34.7) 33 (10.6) 15 (4.8) 10 (3.2) 52 (16.7) 6 (1.9) 4 (1.3) 0
Respiratory support None Only LFNC oxygen therapy Only HFNC oxygen therapy NIV IMV	10 (3.2) 123 (39.5) 87 (28) 87 (28) 4 (1.3)
PICU	100 (32.2)
Length of stay (days)	5 [3–8]

Categorical variables are expressed as numbers and percentage, and continuous variables as median and interquartile range [IQR]

*HFNC* High-flow nasal cannula oxygen therapy, *NIV* Non- invasive ventilation, *IMV* Invasive mechanical ventilation, *PICU* Paediatric Intensive Care Unit

*Bacterial infection: clinical symptomatology and positive blood, urine or tracheal aspirate cultures

^a^Human Coronavirus type 229E, HKU1, OC43 and NL63. ^b^ Influenza A, A/H1, A/H1 - 2009, A/H3 and B

### Outcomes of RSV-associated LRTI hospitalisations

**Fig. 2:**
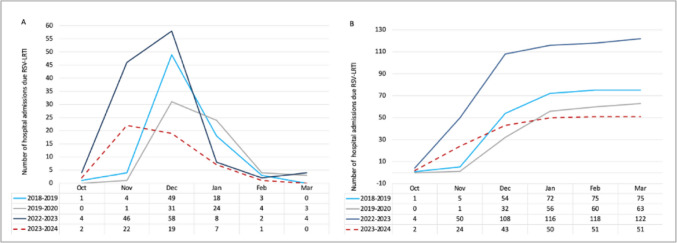
**A**. Monthly distribution of total hospitalisations for RSV-associated LRTI by epidemic season. **B**. Number of cumulative cases (hospitalisations) of RSV-associated LRTI by epidemic season

In the period preceding the pandemic, 75 and 63 admissions occurred during the 2018–2019 and 2019–2020 epidemic seasons, respectively. In contrast, during the postpandemic season, 122 patients were admitted for RSV-LRTI, which is indicative of a larger epidemic outbreak. During the period of administration of nirsevimab, there were 51 hospitalisations, representing a 26.1% (95% CI 15–37.2) reduction in comparison with the average number of admissions in the prepandemic period and a 58.2% (95% CI 49–67.4) reduction in comparison with the postpandemic period (see Fig. 2).

**Fig. 3:**
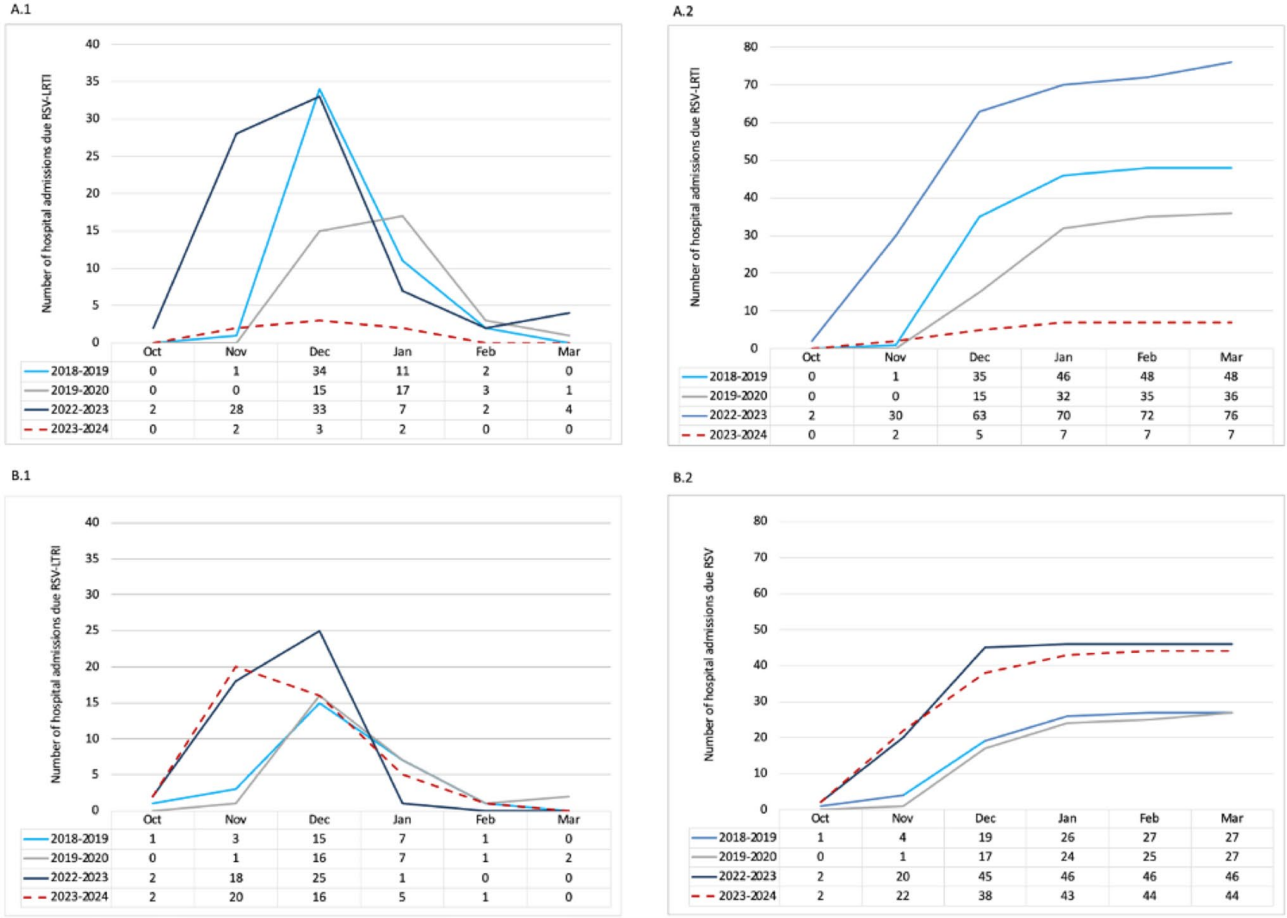
Monthly distribution of hospitalisations (**A.1**) and number of cumulative cases of RSV-associated LRTI by epidemic season in infants aged less than 6 months (**A.2**). Monthly distribution of hospitalisations (**B.1**) and number of cumulative cases of RSV-associated LRTI by epidemic season in children aged 6 months to 5 years (B.2)

The impact of nirsevimab on the reduction of hospitalisations was predominantly observed in infants under six months of age, with only seven hospitalisations due to RSV-LRTI, representing an 83.3% (95% CI 70.9–95.8) decline in admissions compared with the prepandemic average and a 90.8% (95% CI 83.6–98) reduction compared with the postpandemic period in this age group. Immunoprophylaxis was administered to four of the seven children under six months of age who were admitted during the final period. All four immunised infants were healthy infants with no known risk factors. The time between nirsevimab administration and the onset of RSV disease was found to vary, with cases ranging from 14 days to 3 months. Conversely, during the nirsevimab period, the frequency of admissions in children older than six months remained consistent with that observed during the 2023–2024 season, although it was slightly higher than that in the prepandemic period. Figure 3 shows the number of monthly admissions for RSV-LRTI during each epidemic season in infants under six months of age and in children aged six months to five years.

A comparison of the demographic and clinical characteristics revealed a significant increase in the median age during the nirsevimab period (15.6 months; IQR 11.1–27.3) compared with the prepandemic period (4 months; IQR 1.6–9.9) and the postpandemic (3.4 months; IQR 1.5–10.6) season (*p* < 0.001). Furthermore, the diagnosis of bronchiolitis was less prevalent during the nirsevimab period (35.3%) than during the other two periods (prepandemic 73.9% and postpandemic 64.9%; *p *< 0.001). Additionally, the use of antibiotics decreased significantly (prepandemic, 31%; postpandemic, 44.3%; nirsevimab, 23.5%; *p* = 0.0015), and no instances of apnoea were observed during the latter period.

Coinfection with other viruses was found in 57.9% of the children in the total population. The most frequent coinfection was RSV and rhino/enterovirus (34.7%), with an increase in this type of coinfection, as well as in the RSV and adenovirus associations during the nirsevimab period.

The median hospital stay was 4 days (IQR 3–6) in the nirsevimab period compared with 6 days (IQR 4–9) in the prepandemic period and 5 days (IQR 3–8) in the postpandemic period (*p* = 0.003). Table 2 presents a comparison of patient characteristics across the three study periods.

**Table 2:** Characteristics of the patients during the three periods studied

	Pre-pandemic seasons (2018–2019; 2019–2020) *n* = 138 (%)	Post-pandemic season (2022–2023) *n* = 122 (%)	Niservimab period (2023–2024) *n* = 51 (%)	*p-value*
Age (months)	4 [1.6–9.9]	3.4 [1.5–10.6]	15.6 [11.1–27.3]	< *0.001*
Infants < 6 months of age	84 (60.9)	76 (62.3)	7 (13.7)	< *0.001*
Infants < 12 months of age	109 (79)	93 (76.2)	18 (35.3)	< *0.001*
Weight (kg)	6.3 [4.6–9.1]	6 [4.4–9.6]	9.7 [8.1–12]	< *0.001*
Sex (male)	73 (52.9)	79 (64.8)	27 (52.9)	*0.119*
Gestational age (weeks)	39 [38–40]	39 [38–40]	39 [38–40]	*0.446*
Prematurity	19 (13.8)	17 (13.9)	5 (9.8)	*0.792*
Comorbid conditions	9 (6.5)	11 (9)	6 (11.8)	*0.485*
Diagnoses Bronchiolitis Bronchitis/bronchospasm Pneumonia Whooping cough	102 (73.9) 30 (21.7) 6 (4.3) 0	79 (64.9) 33 (27) 9 (7.4) 1 (0.8)	18 (35.3) 24 (47.1) 9 (17.6) 0	< *0.001*
Apnoea	13 (9.4)	5 (4.1)	0	*0.029*
C-reactive protein > 60 mg/L (n = 263)	22 (18.2)	24 (26.7)	7 (14)	*0.148*
Sepsis	6 (4.3)	6 (4.9)	0	*0.329*
*Bacterial infection	15 (10.9)	10 (8.6)	2 (3.9)	*0.351*
Antibiotic therapy	43 (31.2)	54 (44.3)	12 (23.5)	*0.015*
Viral coinfection	72 (52.2)	73 (59.8)	35 (68.6)	*0.108*
Viral Coinfections RSV-Rhino/enterovirus RSV-Coronavirus^a^ RSV-Bocavirus RSV-Parainfluenza 1- 4 RSV-Adenovirus RSV-Influenza^b^ RSV-Metapneumovirus	38 (27.5) 20 (14.5) 15 (10.9) 6 (4.4) 13 (9.4) 3 (2.2) 1 (0.7)	45 (36.9) 11 (9) 0 4 (3.3) 22 (18) 1 (0.8) 1 (0.8)	25 (49) 2 (3.9) 0 0 17 (33.3) 2 (3.9) 2 (3.9)	*0.018* *0.087* < *0.001* *0.358* < *0.001* *0.335* *0.285*
Respiratory support None LFNC oxygen therapy HFNC oxygen therapy NIV IMV	7 (5.1) 47 (34.1) 41 (29.7) 40 (29) 3 (2.2)	0 51 (41.8) 33 (27) 38 (31.1) 0	3 (5.9) 25 (49) 13 (25.5) 9 (17.6) 1 (2)	*0.028*
PICU	48 (34.8)	41 (33.6)	11 (21.6)	*0.204*
Length of stay (days)	6 [4–9]	5 [3–8]	4 [3–6]	*0.003*

Categorical variables are expressed as numbers and percentage, and continuous variables as median and interquartile range [IQR]

*HFNC* High-flow nasal cannula oxygen therapy, *NIV* Non- invasive ventilation, *IMV* Invasive mechanical ventilation, *PICU* Paediatric Intensive Care Unit

*Bacterial infection: clinical symptomatology and positive blood, urine or tracheal aspirate cultures

^a^Human Coronavirus type 229E, HKU1, OC43 and NL63

^b^nfluenza A, A/H1, A/H1 - 2009, A/H3 and B

### Outcomes of PICU admissions due to RSV-associated LRTI

A total of 100 children (32.2%) were admitted to the PICU for severe RSV-LRTI. Fifty-six percent of the patients were transferred from other hospitals within the same health area. The indications for transfer to the PICU were as follows: acute respiratory failure that had not improved despite medical treatment or high-flow nasal cannula (HFNC) oxygen therapy, progressive dyspnoea and hypoxaemia, hypercapnia with acidosis, apnoea or haemodynamic instability. In addition to the above criteria, children were admitted to the PICU from the paediatric ward of our centre in cases of worsening or no improvement despite treatment with HFNC oxygen therapy with a maximum flow rate of 2 L/kg/min in infants up to 12 months of age (10–12 kg of weight) and 1 L/kg/min (maximum 30 L/min) for all other children, with a maximal fraction of inspired oxygen (FiO2) of 40% to achieve an SpO2 between 94 and 97%. In the PICU, 8% of patients continued with HFNC oxygen therapy, while 87% underwent to non-invasive ventilation (NIV): 7% continuous positive airway pressure (CPAP), 25% bilevel positive airway pressure (BLPAP) and 54% used both modalities. The choice of NIV modality (CPAP or BLPAP) or the continuation of HFNC oxygen therapy was determined by the physician responsible for the patient. Four patients required invasive mechanical ventilation (IMV). The indications for IMV were documented as follows: hypoxaemia, hypercapnia, apnoea and severe respiratory distress with signs of imminent respiratory exhaustion. There was no statistically significant difference in the use of ventilatory support between the study periods (p = 0.437).

In the period preceding the pandemic, there were 24 admissions for RSV-LRTI per epidemic season. In contrast, during the postpandemic period, there were 41 admissions for this condition. During the nirsevimab period, there were only 11 PICU admissions for this cause, representing a 54.2% reduction (95% CI 32.2–76.2) compared with the mean number of admissions in the prepandemic period and 73.2% (95% CI 58.4–88) compared with the number of PICU admissions in the postpandemic period.

The group of infants under six months of age presented a significant reduction in PICU admissions, with decreases of 73.3% (95% CI 44.9–92.2) and 87.9% (95% CI 71.8–96.6) compared with those in the pre- and postpandemic periods, respectively. Of the four infants under six months of age admitted to the PICU, three received immunoprophylaxis with nirsevimab. No statistically significant differences were observed in the PICU length of stay. Table 3 shows the characteristics of the children admitted to the PICU for severe RSV-LRTI during the three study periods.

**Table 3:** Characteristics of patients admitted to PICU comparing the three study periods

	Pre-pandemic seasons (2018–2019; 2019–2020) *n* = 48 (%)	Post-pandemic season (2022–2023) *n* = 41 (%)	Niservimab period (2023–2024) *n* = 11 (%)	*p-value*
Age (months)	2 [1.2–12.7]	1.6 [1–4.8]	16.7 [2.5–31]	*0.016*
Infants < 6 months of age	30 (62.5)	33 (80.5)	4 (36.4)	*0.016*
Weight (kg)	5 [3.8–8.9]	5 [3.9–7.3]	7 [5.5–10.3]	*0.195*
Sex (male)	26 (54.2)	31 (75.6)	7 (63.6)	*0.105*
Gestational age (weeks)	39 [37–40]	38 [38–39]	39 [38–40]	*0.196*
Prematurity	7 (14.6)	8 (19.5)	1 (9.1)	*0.732*
Comorbid conditions	4 (8.3)	5 (12.2)	3 (27.3)	*0.233*
Diagnoses Bronchiolitis Bronchitis/bronchospasm Pneumonia	36 (75) 10 (20.8) 2 (4.2)	34 (82.9) 5 (12.2) 2 (4.9)	4 (36.4) 5 (45.5) 2 (18.2)	*0.026*
Apnoea	10 (20.8)	3 (7.3)	0	*0.108*
C-reactive protein > 60 mg/L	10 (21.7)	15 (42.9)	2 (18.2)	*0,077*
Sepsis	4 (8.5)	6 (16.2)	0	0.355
Antibiotic therapy	19 (41.3)	22 (53.7)	5 (45.5)	0.414
Viral coinfection	29 (60.4)	23 (56.1)	7 (63.6)	0.879
Viral Coinfections RSV-Rhinovirus/enterovirus RSV-Coronavirus^a^ RSV-Bocavirus RSV-Parainfluenza 1- 4 RSV-Adenovirus RSV-Influenza^b^ RSV-Metapneumovirus	15 (31.3) 8 (16.7) 7 (14.6) 0 5 (10.4) 2 (4.2) 1 (2.1)	18 (43.9) 3 (7.3) 0 1 (2.4) 5 (12.2) 0 0	6 (54.5) 0 0 0 4 (36.4) 0 0	*0.254* *0.237* *0.017* *0.525* *0.103* *0.603* *1*
Respiratory support HFNC oxygen therapy NIV IMV	4 (8.3) 40 (83.3) 3 (6.3)	3 (7.3) 38 (92.7) 0	1 (9.1) 9 (81.8) 1 (9.1)	*0.437*
Duration of respiratory support (days)	4.3 [2–5.6]	3 [2–4]	3 [2–4]	*0.362*
Length of PICU stay (days)	5 [2.3–6]	4 [3–5]	3 [3–5]	*0.456*
Length of stay (days)	9.5 [7–13]	8 [7–11]	5 [5–9]	*0.089*

Categorical variables are expressed as numbers and percentage, and continuous variables as median and interquartile range [IQR]

*HFNC* High-flow nasal cannula oxygen therapy, *NIV* Non- nvasive ventilation, *IMV* Invasive mechanical ventilation, *PICU* Paediatric Intensive Care Unit

*Bacterial infection: clinical symptomatology and positive blood, urine or tracheal aspirate cultures

^a^Human Coronavirus type 229E, HKU1, OC43 and NL63

^b^Influenza A, A/H1, A/H1 - 2009, A/H3 and B

## Discussion

Following the implementation of immunoprophylaxis with nirsevimab during the 2023–2024 epidemic season, a reduction in hospitalisations and PICU admissions due to RSV-LRTI in children under five years of age was observed in a health area in Spain. This effect was especially relevant in the group of infants younger than six months, who have been the main target of this passive immunisation strategy.

Two randomised clinical trials involving healthy infants, most of whom were born at term, demonstrated that nirsevimab administration reduced RSV-related hospitalisation [6, 19, 20]. The efficacy of nirsevimab in preventing RSV-LRTI hospitalisation has been corroborated by data from studies conducted in real-world settings that have recently been published [21–26]. The interim analysis results from the NIRSE-GAL study (Spain) demonstrated that nirsevimab exhibited an efficacy of 82% (95% CI 65.6–90.2) in reducing RSV-LRTI hospitalisations. The monoclonal antibody demonstrated an efficacy of 86.9% (95% CI 69.1–94.2) in preventing severe illness requiring oxygen in infants born during the epidemic season and in infants under six months of age at the start of the immunisation campaign [25]. Similarly, following the introduction of passive immunisation with nirsevimab during the 2023–2024 season in neonates and infants born from 1 January 2023, a reduction in RSV hospitalisations was observed in Luxembourg (preliminary analysis) [23].

The present study analysed data from the entire 2023–2024 epidemic season, with findings that align with the preliminary data published in population-based studies conducted in real-world settings [23, 25, 26]. The findings revealed that universal passive immunisation with nirsevimab resulted in a decrease in hospitalisations and PICU admissions due to RSV-LRTI, particularly among infants under six months of age. In comparison with the 2022–2023 season, there was a notable decrease in the incidence of hospitalisations and PICU admissions within this age group, with a 90.8% reduction in hospitalisations and an 87.9% reduction in PICU admissions. The high rate of vaccination coverage in the health area may have contributed to a greater reduction in hospitalisations. No significant differences were identified in the group of children aged six months to five years.

In view of the exceptional 2022–2023 epidemic season, which featured a significant increase in hospitalisations and intensive care requirements [27], it was deemed essential to compare the data obtained during the nirsevimab period with those of the two seasons preceding the COVID- 19 pandemic. The findings also revealed a notable reduction in hospitalisations and PICU admissions among infants under six months of age.

In Spain, RSV infection has been monitored by the Acute Respiratory Infections Surveillance System (SiVIRA) since the 2021–2022 season. The SiVIRA primary care sentinel surveillance network reported an overall RSV test positivity of 5.3% in the 2022–2023 season and 4.3% in the 2023–2024 season, reflecting a slight decrease in virus circulation during the last epidemic season [28, 29]. However, the number of hospitalisations due to RSV-LRTI in children at our centre fell by almost 60%, a reduction that we consider significant and not justified by the circulation being slightly lower during the previous season.

The decrease in the frequency of bronchiolitis observed during the last period is indicative of an important decline in primary RSV infection rates among young infants. Acute bronchiolitis is the primary cause of LRTI-related hospitalisation in children under one year of age, and it can result in severe acute complications such as apnoea and respiratory failure [30]. Furthermore, studies have indicated that infants with severe bronchiolitis who require hospitalisation may develop asthma during childhood and adulthood. This can have a significant effect on their quality of life, leading to frequent visits to health care services [31, 32]. In recent years, the majority of infants hospitalised with RSV-associated bronchiolitis were born at full term and did not have known risk factors [2]. Consequently, nirsevimab provides protection to this group of children who were previously vulnerable to severe RSV infection. It would be beneficial to evaluate the indirect effect that universal immunoprophylaxis with nirsevimab could have on childhood asthma rates, which should be considered in future studies.

In our cohort, we observed a shift in the age profile of infants hospitalised due to RSV during the nirsevimab period. Specifically, 65% of patients were over one year old, in contrast to previous seasons, where 75% to 80% were under one year old. This finding has also been reported in other series [22, 25, 33]. Older age at the time of RSV infection is associated with less severe illness, a lower risk of hospitalisation, and fewer short- and long-term consequences [32]. The older age of patients in the present study may explain the absence of apnoeic episodes, the reduced use of antibiotics, the lower number of admissions to the PICU, and the shorter length of hospital stay during the nirsevimab period.

Notably, four out of seven infants under six months of age who were hospitalised (57.1%) had received nirsevimab, and three of them were admitted to the PICU. These findings differ from those of other published studies, where the majority of patients hospitalised with RSV-LRTI had not been previously immunised with nirsevimab [18, 21]. The high rate of vaccine coverage in our setting may be a contributing factor. Additionally, two immunised patients exhibited coinfection with rhino/enterovirus, and it was hypothesised that this may have exacerbated the clinical picture and led to hospitalisation. Costa et al. reported that, compared with RSV alone, coinfection with entero-rhinovirus is associated with more severe lower respiratory tract symptoms and a higher rate of hospitalisation [34]. However, cases of entero-rhinovirus should be interpreted with caution, as viral RNA has been found in nasopharyngeal samples 4–5 weeks after infection and is detected in 16–38% of asymptomatic children [35]. We believe that other potential factors that could explain the failure of immunisation include the partial efficacy of the treatment in the face of intense virus exposure, the decrease in antibody levels as the epidemic season progresses, or even errors in the administration of the injection. However, further investigations are needed to substantiate these hypotheses. In a recent study, Wilkins and colleagues analysed over 5,600 RSV fusion protein sequences from across the globe between 2015 and 2021. Their findings revealed that certain viruses acquired mutations at the nirsevimab binding site, thereby reducing the ability of monoclonal antibodies to neutralise the virus. These resistant mutations account for less than 1% of all circulating viruses [36]. While their frequency remained consistent from one season to the next, it is unclear whether these resistant viruses could increase over time with the widespread use of nirsevimab [37].

The increase in coinfections with rhinovirus/enterovirus and adenovirus in our population during the recent period is a cause for concern, as it may indicate an increase in the prevalence of other respiratory viruses beyond RSV. The reasons for this increase are unknown. Nevertheless, close monitoring of various circulating pathogens in the coming years is crucial.

Spain was among the first European countries to integrate nirsevimab into a national vaccination programme, a strategy that was not without controversy owing to the absence of cost-effectiveness studies to support its widespread administration. Although the results of its universal use in different areas are already emerging, there is a paucity of real-world studies. Consequently, our research provides specific and detailed information on the effectiveness of nirsevimab in a population of children under five years of age. This includes a comparison of the diagnoses, characteristics, and complications of patients, as well as an analysis of epidemiological patterns and associations with other viral agents during different epidemic seasons, including the prepandemic era.

Importantly, this study has several limitations that should be taken into account when the results are considered. These include a single-centre design with a modest sample size, which means that the results reflect only the situation in a specific geographic health area. However, our results are similar to those in other geographic areas in Spain and Europe, which supports the effectiveness of this preventive strategy in different populations.

## Conclusions

In conclusion, the implementation of the universal passive immunisation strategy with nirsevimab resulted in a reduction in hospitalisations and PICU admissions for RSV-LRTI in a Spanish health area. Additionally, there was a shift in the age profile of hospitalised patients and a shorter length of hospital stay. These findings are encouraging, and it is anticipated that in the coming years, nirsevimab prophylaxis will mitigate the significant burden on health care services during the winter season. Nevertheless, close epidemiological surveillance in the coming years and economic evaluation studies that substantiate the reasonable cost-effectiveness of this preventive measure will be necessary.

## Data Availability

No datasets were generated or analysed during the current study.
